# Trends in Anemia Prevalence Among Indian Women Using Revised WHO Hemoglobin Cutoffs: Insights From Repeated Cross-Sectional Surveys (1998–2019)

**DOI:** 10.1155/anem/5214630

**Published:** 2025-07-02

**Authors:** Anuj Kumar Pandey, Diksha Gautam, Benson Thomas M., Sutapa Bandyopadhyay Neogi

**Affiliations:** ^1^Department of Health Systems and Implementation Research, International Institute of Health Management Research, New Delhi, India; ^2^Institute for Population and Social Research, Mahidol University, Salaya, Thailand; ^3^School of Public Health, SRM Institute of Science and Technology, Chennai, India

**Keywords:** anemia, hemoglobin, severe anemia, sustainable development goals

## Abstract

**Introduction:** This study reanalyzes the data from India based on revised cutoffs on hemoglobin (Hb) as updated by World Health Organization (WHO) to inform policy decisions.

**Methods:** The study analyzes change in prevalence of anemia among pregnant women in different trimesters and nonpregnant women in India using data from nationally representative survey (1998-1999 to 2019–2021). Changes in mean Hb concentration and in anemia prevalence using revised Hb cutoffs were analyzed. Mann–Kendall (MK) test was utilized to estimate trend coefficient (*τ*) with significance to quantify change in anemia prevalence. State-wise anemia prevalence was calculated, categorizing states as controlled, emerging hotspots, hotspots, and arresting momentum, considering 40% prevalence cutoff as severe public health concerns.

**Results:** For 2019–2021, anemia prevalence during pregnancy decreased from 52.2% as per the previous cutoff to 47.1% as per the revised cutoff. As per revised cutoffs, MK test revealed reduction in anemia trends (*τ* = 0.333, *p*=0.734). This decline was most pronounced in the third trimester, where severe anemia dropped by 51.3% from 1998–1999 to 2019–2021. While mild and moderate anemia showed little change over two decades, moderate anemia in the third trimester declined by 3.67%. Reductions in severe anemia were noted across trimesters. States identified as emerging hotspots require urgent, targeted interventions due to persistently high or rising anemia rates.

**Conclusion:** This study highlights a decline in anemia prevalence, particularly in severe cases during pregnancy. Anemia reduction efforts must rely on country-specific data, especially on the Hb distribution against the background of ferritin level, hemoglobinopathies, prevalence of infections, and inflammations in the population.

## 1. Introduction

Anemia is a condition usually identified when the hemoglobin (Hb) concentration drops below a defined threshold [1]. From a functional perspective, anemia exists when circulating red blood cells are inadequate to sufficiently deliver oxygen to peripheral tissues [2]. Anemia continues to be a global public health problem, and women of reproductive age are at a greater risk [3, 4] with serious implications for women's health and their productivity. The cause of anemia can be due to both nutritional and non-nutritional factors, with iron deficiency being the most common and a leading contributor to the global burden of disease, particularly affecting people in low- and middle-income countries [5].

As per World Health Organization (WHO), the global prevalence of anemia in women of reproductive age group was 29.9%, and in nonpregnant women of reproductive age, it was 29.6% in 2019. However, prevalence was relatively higher in pregnant women at 36.5% [6]. Recent data show that around 45.2% pregnant women and 39.5% nonpregnant women have anemia in low- and middle-income countries [7]. The WHO Global Nutrition Targets is committed to a 50% reduction in anemia in women of reproductive age by 2025 [8].

Burden of anemia status in India continues to be a cause of concern. Despite several intensive programs by Government of India, the country has witnessed a fluctuating trend in the prevalence of anemia. The recent National Family Health Survey (NFHS) survey findings have shown an upward trajectory of anemia in India from 54.1% in 2015–2016 to 59.1% in 2019–2021 [9]. Controlling the burden of anemia has been accorded high public health priority as evident from several initiatives undertaken to address the problem [10]. Since 1970, when the National Anemia Control Program was launched, the country has witnessed renewed focus through changes in programs and policies over the past several years. Complying with the targets of POSHAN Abhiyaan and National Nutrition Strategy, government has launched the Anemia Mukt Bharat program in year 2018 with an objective to reduce prevalence of anemia by 3% points per year among children, adolescents, and women in the reproductive age group (15–49 years), between the year 2018 and 2022 [11, 12]. However, the persistent high prevalence of anemia among Indian women despite insistent efforts is a cause of concern.

Hb or hematocrit concentration tests for anemia are the most widely used screening techniques to assess the presence of iron deficiency anemia in a population [13]. These measurements can be performed in the field, are rather easy and inexpensive, and levels below a predetermined cutoff point identify or suggest the possibility of anemia. The current cutoff value defining iron deficiency anemia according to Anemia Mukt Bharat Guidelines of Ministry of Health and Family Welfare (MoHFW) for nonpregnant women with 15 years of age and above is 11–11.9 g/dL (mild); 8–10.9 g/dL (moderate); and < 8 (severe). For pregnant women, the cutoff value is 10–10.9 g/dL (mild); 7–9.9 g/dL (moderate); and < 7 g/dL (severe) [14]. Discussions have been underway to consider revising the cutoffs based on emerging evidence [15, 16]. Hb levels have been noted to naturally fluctuate during pregnancy due to hemodilution, especially during the second trimester when plasma volume is greater than the red cell mass [17]. Considering the range of other complexities across different populations, WHO has recently (2024) released an updated guideline [18] on Hb cutoffs to define anemia in individuals and populations to provide updated, locally adaptable, clear, evidence-informed normative statements on the use of Hb concentrations to assess anemia and on best approaches in its measurement in individuals and populations. The new trimester-specific classification aims to more accurately reflect these physiological adaptations and improve the identification and management of true anemia in pregnant women [17]. The revised Hb cutoff for pregnant women is categorized by trimesters: for first trimester, ≥ 11 g/dL (no anemia), 10–10.9 g/dL (mild anemia), 7–9.9 g/dL (moderate anemia), and < 7 g/dL (severe anemia); for second trimester, ≥ 10.5 g/dL (no anemia), 9.5–10.4 g/dL (mild anemia) 7–9.4 g/dL (moderate anemia), and < 7 g/dL (severe anemia); and for third trimester, ≥ 11 g/dL (no anemia), 10–10.9 g/dL (mild anemia), 7–9.9 g/dL (moderate anemia), and < 7 g/dL (severe anemia). The Hb cutoff for nonpregnant women aged 15–45 years is same as of Anemia Mukt Bharat Guidelines [18].

It therefore becomes imperative to reanalyze the data based on the revised cutoffs to inform policy decisions. The current study aims to analyze the change in prevalence of anemia as per the recent WHO Hb cutoffs among pregnant women in different trimesters and nonpregnant women in India from 1998-1999 to 2019–2021. The objectives of the study are as follows:

1. To analyze change in mean Hb concentration for selected sample population in India from 1998-1999 to 2019–2021.
2. To analyze the change in the prevalence of anemia using revised Hb cutoffs in India.
3. To categorize states based on anemia prevalence using revised cutoffs and rate of change in Anemia.

## 2. Methods

### 2.1. About the Revised Guideline

The WHO in its recent publication [18] introduced a revised guideline for measuring Hb and defining anemia. The objective of the guideline is to “provide updated, clear, evidence-informing normative statements on the use of Hb concentrations to assess anemia and on the best approaches in its measurement in individuals and populations.”

The recent cutoffs for defining anemia have been developed based on a commissioned analysis of Hb levels from healthy populations and systematic reviews following protocols laid down in standard guideline book of WHO [19]. This guideline revises and supplements previous recommendations found in five WHO publications issued between 1968 and 2005 [20–24], compiled during 2011 [25]. The guideline [18] provides a thorough discourse on the rationale and methodology employed during development of the revised cutoff. Despite having revised Hb cutoffs for various age groups of children, women, and pregnant women, our study's focus limits examination solely to nonpregnant and pregnant women (Table 1).

### 2.2. Data Source and Sample

This study used data on Hb levels from India's Demographic and Health Survey (DHS) named as the NFHS [26]. India has undertaken five rounds of nationally representative sample surveys spanning from 1992 to 1993, 1998 to 1999, 2005 to 2006, and 2015 to 2016, with the most recent being NFHS-5, conducted from 2019 to 2021. NFHS provides comprehensive data on various subjects including reproductive healthcare, fertility, family planning, etc. Information on selected chemical, anthropometric and biochemical parameters (CAB) from children aged 0–71 months, women aged 15–49 years, and men aged 15–54 years were also collected from some of the rounds of NFHS including data on the Hb concentration to assess anemia prevalence among various age groups. NFHS employs a two-stage stratified random sampling method for sample selection in each round with a total of 90,303 eligible women aged 15–49 years interviewed during 1998–1999, 124,385 during 2005–2006, 699,686 during 2015–2016, and 699,686 eligible women during 2019–2021. Notably, all these survey rounds achieved an overall response rate exceeding 97%. Women were informed, and their consent was obtained before sample collection (Table 2).

#### 2.2.1. Method Used for Anemia Estimation

NFHS follows a standard protocol of selecting and training health investigators for collecting blood specimens from eligible respondents for anemia testing. For all the selected rounds for this analysis, blood samples were obtained for Hb testing only after ensuring consent from the eligible respondents. During every survey round, a minute amount of capillary blood, obtained via finger prick (for eligible women), was utilized to determine blood Hb concentration using the HemoCue Hb 201+ analyzer, which promptly provides on-site blood Hb concentration readings [27]. Consistent utilization of capillary blood and the HemoCue Hb 201+ analyzer across NFHS surveys facilitates the tracking of changes in anemia prevalence over time. It is noteworthy that anemia levels were estimated after adjusting for altitude and smoking in accordance with DHS protocols [28].

### 2.3. Variables

We have analyzed data on Hb concentration (in *gm/dL*) to meet the objective. Other variables, namely, currently pregnant women in different trimesters and nonpregnant women, were also used as an exposure variable for the analysis.

### 2.4. Statistical Analysis

This study attempts to analyze the prevalence of anemia among pregnant women in different trimesters and nonpregnant women from 1998–1999 to 2019–2021. We have furthermore evaluated the change in the prevalence of anemia at different Hb cutoffs to demonstrate actual change in the pattern of anemia in India.

We have analyzed the change in mean Hb concentration during the study period for the selected study sample population. We have also plotted it graphically to display the percentage distribution of women with different Hb concentrations over different rounds of NFHS. Utilizing the data on Hb concentration, we have analyzed the prevalence of anemia severity at the recommended revised Hb cutoffs in individuals. We have used the cutoffs stated by WHO as no anemia, mild, moderate, and severe anemia to categorize each study sample. To present percentage change, we have calculated relative change in the prevalence of anemia from 1998–1999 to 2019–2021 to identify categories that need intervention.

The formula used for calculation of relative change is *y*=(*x*2 − *x*1/*x*1)∗100, where *y* is the relative change, *x*1 is the initial year value, that is, prevalence in 1998–1999, and *x*2 is the recent year prevalence, that is, prevalence during 2019–2021. Also, to quantify the change in prevalence of anemia from 1998–1999 to 2019–2021 among pregnant and nonpregnant women, Mann–Kendall (MK) test was utilized to estimate trend coefficient (*τ*) with significance. The MK test was used to analyze trends in anemia prevalence due to its suitability for datasets with limited time points and its ability to provide statistical evidence when parametric assumptions are unmet. While nonparametric tests like MK inherently have lower power, they remain appropriate for this context. The intent is to quantify these changes rather than solely assessing statistical significance.

In order to understand the actual level for decline in Hb concentration, we have again categorized the Hb concentration and analyzed the prevalence of anemia in different study samples. Later prevalence of anemia among pregnant women was analyzed both at the previous cutoff of 11.0 gm/dL and at cutoff with 11.0 gm/dL for women in first and second trimesters and 10.5 gm/dL in the second trimester.

Lastly, we have calculated state-wise prevalence of anemia among women of reproductive age (≥ 12.0 gm/dL) and pregnant women (11.0 gm/dL for women in first and third trimesters and 10.5 gm/dL in the second trimester) with the updated cutoff of and rate of change over the year. Dadar Nagar haveli, Daman Diu, and Ladakh were removed from this analysis due to data unavailability. For percentage change, 2019–2021 is considered as *x*2, whereas the extreme last year was taken as reference for *x*1 (for some states/UT, 1998–1999 was not the extreme last year). Based on the rate of change and prevalence, states were classified as controlled, emerging hotspot, hotspot, and arresting momentum. A prevalence of 40% was taken as the cutoff for this classification where controlled indicated low prevalence and decreasing rate of change, emerging hotspot indicated low prevalence but high rate of change, hotspots are states with high prevalence and high rate of change over the year, whereas states in arresting momentum indicate high prevalence but low rate of change. A prevalence of 40% was considered as the cutoff as below this, anemia ceases to be a severe public health problem.

### 2.5. Ethics Approval

All data used for analysis were accessed from DHS website post registration. The available data are anonymized and available in public domain thus have no ethical implication [29].

## 3. Results

### 3.1. Magnitude of Change in Prevalence of Anemia (1998–2021) due to Change in Cutoff Values

**Figure 1:**
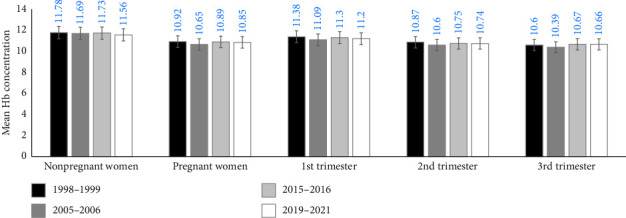
Mean hemoglobin concentration with 95% CI across 4 phases of NFHS.

An analysis of mean Hb concentration from several rounds of NFHS, displays that the mean (Sd) Hb concentration of the study sample did not observe much change over the years in India. Mean (Sd) Hb concentration among nonpregnant women age Group 15–49 years shifted from 11.78 (1.90) during 1998-1999 to 11.56 (1.63) during 2019–2021 (Figure 1). The overall mean Hb concentration for the women in second and third trimesters ranges below 11.0 gm/dL, whereas during the first trimester, it ranged above 11.0 gm/dL but lower than 11.50 gm/dL.

**Figure 2:**
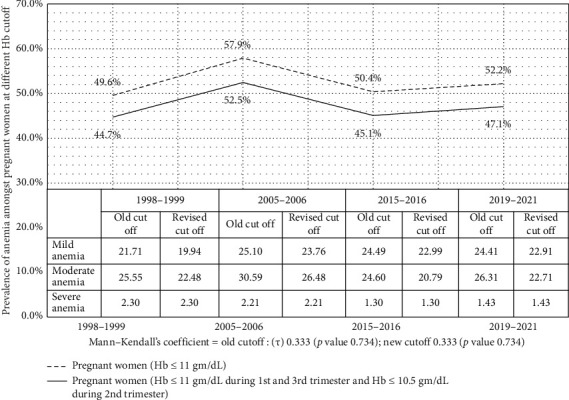
Prevalence of anemia during pregnancy at two different cutoffs from 1998-1999 to 2019–2021 (India).

On comparison with the old guidelines revised guidelines cutoff shows a reduction in prevalence in anemia during pregnancy from 52.2% to 47.1% during 2019–2021 (Figure 2). MK test result shows a trivial change in anemia prevalence trend for as per revised cutoff (*τ* = 0.333; *p* value 0.734) as also for prevalence as per old cutoff (*τ* = 0.333; *p* value 0.734). The revised cutoff results show a notable variance in the prevalence of anemia over the years, showing an approximate 5%–6% difference from old cutoff. Figure 2 also shows that mild anemia consistently accounted for the lowest proportion, while moderate anemia remained the most prevalent across all periods. Severe anemia showed minimal variation, staying relatively stable. The revised cutoff, which considers different thresholds for trimesters, slightly lowered prevalence estimates compared to the old cutoff.

### 3.2. Trend of Anemia Prevalence—As Per Revised Cutoff

Table 3 presents the relative change in prevalence of anemia over the years as per the revised Hb cutoffs given by WHO. Findings suggest that there is a significant reduction in the overall prevalence of “no anemia” in all the categories, that is, nonpregnant women and pregnant women in different trimesters. This change ranges from −5% during first trimester of pregnancy to −11.4% in nonpregnant women. Overall 42.75% of nonpregnant women are nonanemic during 2019–2021, whereas this percentage is as high as 59.3% during the first trimester of pregnancy and 55.9% during the second trimester of pregnancy. The table also presents that there is significant decline in the prevalence of severe anemic women in India. The decline ranges between −51.3% among women in the third trimester of pregnancy and 26.2% among nonpregnant women in India.

All the other trimesters analyzed show a concomitant increase in the prevalence of moderate and mild anemia. It is pertinent to note that with time, there has been a shift with more women entering moderate, mild and no anemia categories.

The change in Hb concentration is more pronounced in the third trimester of pregnancy. During 2019–2021, 1.6% women had severe anemia which has declined by −51.3% from 3.3% during 1998–1999. Additionally, it is worth mentioning that moderate anemia throughout all trimesters has exhibited a more pronounced change compared to mild anemia. MK test resulted in similar trends for the first and second trimesters of pregnancy for all kinds of anemia. Severe anemia among third trimester of pregnant women exhibited a declining trend (τ = −0.667, p-value = 0.308).

Distribution of Hb concentration (Supporting File 1) presents that there is a shift in the overall Hb concentration over the year with the exception of women during third trimester which presented a significant shift in the overall Hb concentration. Shift along with the clustering or concentration of the percentage of women around Hb concentration is also evident from the graphs.

### 3.3. Statement on Public Health Significance of Anemia in India As Per the Latest Hb Cutoff

The recent guidelines have retained the previous classification of anemia prevalence in population to define its public health significance [18]. An overall prevalence of 40% or above qualifies as a severe public health problem, whereas 20.0%–39.9% would be classified as moderate, 5%–19.9% would be classified as mild, and less than 4.9% would be categorized as no anemia. The overall prevalence is close to 47%. Even if we disregard the variations that might crop up due to measurement and methodological issues, it would be prudent to note that we may cross the threshold in the near future.

### 3.4. Comparison of Prevalence of Anemia

**Figure 3:**
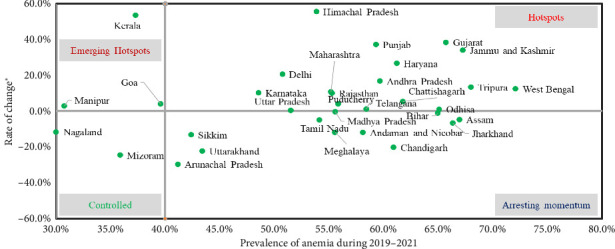
State-wise change in the anemia prevalence from 1998-1999 to 2019–2021 among nonpregnant women (15–49 years).

**Figure 4:**
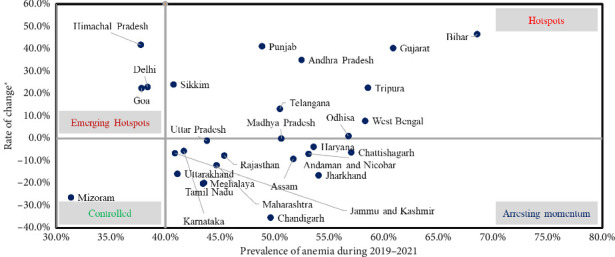
State-wise change in the anemia prevalence from 1998–1999 to 2019–2021 among pregnant women.

To further assess the state-wise change in the anemia prevalence among nonpregnant women and pregnant women, we have analyzed prevalence and rate of change of anemia prevalence from 1998-1999 to 2019–2021. We have classified the states in four different quadrants as presented in Figures 3 and 4 to present states requiring urgent intervention. Supporting File 2 provides state-wise point estimates of anemia prevalence among pregnant and nonpregnant women with 95% CIs for each survey round.

States falling into the category of emerging hotspots and hotspots need urgent state-specific intervention where the prevalence is either very high or low with a high rate of change, that is, the prevalence is increasing between 0% and 60%. Among nonpregnant women, Kerala, Goa, and Manipur demand urgent call for action (Figure 3).

Figure 4 provides a snapshot of states where focus should be diverted as in case of some states/UTs where the rate of change is highest irrespective of prevalence of anemia among pregnant women as depicted in the hotspot zone.

## 4. Discussion

An analysis of the prevalence of anemia during pregnancy based on revised WHO cutoffs using data from four rounds of a nationally representative survey demonstrates reduction in anemia prevalence from 52.2% to 47.1% during 2019–2021. The change is most pronounced during the third trimester that shows a decline in severe anemia from 3.3% in 1998-1999% to 1.2%. There is a demonstrated reduction in severe anemia across the spectrum of women in the reproductive age group.

The analysis was planned in response to prolonged deliberations regarding stagnancy in the prevalence of anemia among pregnant women in India despite focused interventions like Anemia Mukt Bharat. This nation-wide program strategically intervenes and plans to alleviate burden of anemia among pregnant and nonpregnant women [30]. Recent studies have shown a dramatic increase in the compliance to the programmatic interventions to accelerate reductions in anemia prevalence [31]. Despite extensive efforts, there has been no notable improvement in the prevalence of anemia in India [15, 31–33]. Dearth of population-based and nationally representative surveys around the diverse clinical etiologies of anemia prompts inquiry into the rationale of programmatic interventions implemented thus far.

Recent updates by WHO [18] on Hb cutoffs is a welcoming move that aims to redefine anemia in individuals and populations to provide updated, locally adaptable, clear, evidence-informed normative statements on the use of Hb concentrations to assess anemia and on best approaches in its measurement in individuals and populations. We have analyzed four rounds of nationally representative DHS data, the only representative survey which provided information on Hb concentrations from women of reproductive age until 2019–2021. One of the other key strengths of our study lies in the consistent utilization of the HemoCue device to measure Hb level [32] across four rounds spanning approximately two decades. The prevailing controversy around venous/capillary blood collection and instruments used for sample collection and analysis is a concern [32, 34–36]. However, DHS in India ensures validity by strong training of the sample collectors and by maintaining uniformity in data collection, instrument usage, and analysis procedures. Given the literature and ongoing debates, it is clear that India lacks an accurate measure of anemia prevalence across all age group. The use of robust statistical techniques to analyze change in the prevalence across years gives strength to our analysis, thereby enhancing its utility for subsequent policy deliberations.

While this study provides valuable insights into anemia trends in India from 1998–1999 to 2019–2021, several limitations must be considered. First, the use of secondary data from the NFHS may introduce reporting biases, as data collection methods and participant self-reporting could vary across survey rounds; however, reported consistencies across rounds might overcome this limitation. Additionally, the revised Hb cutoffs used in this study, while standardized, may not fully account for other factors like ferritin levels, hemoglobinopathies, or infection status, which could influence anemia prevalence. The magnitude and direction of bias could vary by region and population subgroup, impacting the generalizability of the findings.

Except for anemia resulting from hemoglobinopathies that may be considered a “disease”, anemia is a quantitative expression of a biological variable, that is, Hb. The distribution of Hb in the target populace remains the guiding principle to identify a suitable cutoff. The revised WHO guidelines have considered 5th percentile considering that it would provide more sensitivity to detect individuals with underlying conditions associated with anemia (e.g. nutritional deficiency, genetic conditions, inflammation, infection), thereby promoting intervention [18]. The trade-off between normality and abnormality on the distribution of Hb in a population is confounded by individual factors such as age, sex, altitude, smoking status, measurement errors, observer's bias, and random variation. However, it must be borne in mind that cutoffs should not only be determined on the basis of statistical consideration but also be supported and interpreted in the light of prognostic significance [37, 38].

Our study noted that there is a significant decline in the anemia prevalence during the third trimester of pregnancy. There is an obvious shift in the distribution of Hb to the right among pregnant women over the past several years. This decline could be attributed to implementation of the programmatic interventions with a focus on pregnant women or to factors stemming from overall development. Also, there is a decline in the severe anemia prevalence across women in different trimesters and among nonpregnant women that is a testimony of the fact that there is a positive trend in Hb levels in the population. Understandably, this has resulted in more women in the mild and moderate anemia category.

Further, our study also evaluated status of states/union territories based on rate of change of anemia and current prevalence of anemia. We noted some states/UTs as emerging hotspots for anemia, like Delhi, Goa, and Himachal Pradesh, for nonpregnant women, whereas Kerala, Goa, and Manipur for pregnant women. On the other side, the majority of the states/UTs fall in the category of hotspot that implies that the prevalence is also high, and rate of change is also high. These could be due to a combination of factors like dietary pattern, socioeconomic factors, access to healthcare, prevalence of other infectious diseases, and cultural and contextual factors. Additionally, differences in the implementation of prevention and control programs, as well as variations in the availability and utilization of healthcare services, could also contribute to the observed disparities in anemia prevalence across different states and union territories.

It is fairly well documented that the relative portion of anemia that is due to iron deficiency globally is likely to be less than half. Petry et al. conducted a meta-analysis of nationally representative surveys from 23 countries (including low, medium, and high human development index) utilizing biomarkers to elucidate prevalence of iron deficiency, iron deficiency anemia, and anemia among women of reproductive age. In these surveys, the proportion of anemia associated with iron deficiency ranged from 2.9% (Georgia) to 74.7% (Oman), with an overall average of 37.0%. Despite large heterogeneity, the analyses suggested that the proportion of anemia associated with iron deficiency is lower than the previously assumed 50% in countries with low, medium, or high human development index ranking [39]. Anemia reduction strategies and programs should be based on an analysis of country-specific data, as iron deficiency may not always be the key determinant of anemia.

Nevertheless, we need to continue our efforts to address the persistent problem of anemia in India. Supplementation of oral iron for a longer duration [40–42], improving adherence to oral therapy, increasing proportion of women who attend at least four ANC visits, provision of good and adequate antenatal care [42–44], and providing intravenous therapy to select women who present with severe anemia or moderate anemia (who do not respond to oral therapy or report late in pregnancy [45]) should be implemented with focused attention. It is worth considering that iron deficiency can also occur in the absence of anemia, when ferritin concentrations are low, but Hb concentrations are adequate [46]. Aggressive attempts to identify and treat anemia must be balanced carefully with the risks of overdiagnosis and overtreatment.

To conclude, the prevalence of anemia among pregnant and nonpregnant women aged 15–49 years, based on WHO revised guidelines, suggests that it has shown a decline over the past 20 years in India. The reduction is notable in severe anemia across the spectrum, especially in the third trimester of pregnancy. Anemia reduction efforts must rely on country-specific data, especially on the Hb distribution against the background of ferritin level, hemoglobinopathies, prevalence of infections, and inflammations in the population.

## Figures and Tables

**Table 1:** Recommended hemoglobin cutoffs (g/L) to define anemia severity in individuals.

Population	Previous: 2011 guideline				Revised: 2024 guideline			
	No anemia	Anemia			No anemia	Anemia		
		Mild	Moderate	Severe		Mild	Moderate	Severe
Nonpregnant women	≥ 120	110–119	80–109	< 80	≥ 120	110–119	80–109	< 80
Pregnant women	≥ 110	100–109	70–99	< 70				
1^st^ trimester	≥ 110	100–109	70–99	< 70	≥ 110	100–109	70–99	< 70
2^nd^ trimester	≥ 110	100–109	70–99	< 70	≥ 105	95–104	70–94	< 70
3^rd^ trimester	≥ 110	100–109	70–99	< 70	≥ 110	100–109	70–99	< 70

**Table 2:** Sample for estimation of anemia prevalence in India (1998–1999 to 2019–2021)^∗^.

Status (weighted) (Hb measured)	Sample (*N*)			
	NFHS-2	NFHS-3	NFHS-4	NFHS-5
Nonpregnant women	74,234	110,827	649,119	656,232
Pregnant women in different trimester at the time of survey				
1st trimester (0–12 weeks)	1511	1541	8990	7525
2nd trimester (13–26 weeks)	2172	2347	12,107	9846
3rd trimester (27 weeks to delivery)	1878	2140	9216	8404

*Note:* Few cases (∼10–40) were missing for hemoglobin examination when pregnancy status was divided by trimester.

^∗^Number of women who gave consent and blood sample collected.

**Table 3:** Prevalence of anemia in India as per the revised cutoffs given by WHO.

Prevalence of anemia in India as per the revised cutoffs	1998–1999 (%)	2005–2006 (%)	2015–2016 (%)	2019–2021 (%)	Relative % change 1998–1999 to 2019–2021 (%)	Mann–Kendall's coefficient (*τ*)	*p*-value
*Nonpregnant women (15–49 years)*							
No anemia (≥ 12.0 gm/dL)	48.27	44.84	46.76	42.75	**−11.44**	**−0.667**	0.308
Mild anemia (11.0–11.9 gm/dL)	21.64	23.81	24.73	25.69	**18.74**	**1**	0.089
Moderate anemia (8.0–10.9 gm/dL)	26.39	27.54	26.03	28.83	**9.23**	**0.333**	0.734
Severe anemia (< 8.0 gm/dL)	3.66	3.80	2.45	2.71	*−26.17*	*−0.333*	0.734

*First trimester of pregnancy (0–12 weeks)*							
No anemia (≥ 11.0 gm/dL)	62.44	55.43	59.40	59.29	**−5.04**	**−0.333**	0.734
Mild anemia (10.0–10.9 gm/dL)	18.52	22.15	22.80	21.56	**16.40**	**0.333**	0.734
Moderate anemia (7.0–9.9 gm/dL)	17.48	20.68	16.80	17.90	**2.42**	**0.333**	0.734
Severe anemia (< 7.0 gm/dL)	1.56	1.73	0.97	1.19	*−23.98*	*−0.333*	0.734

*Second trimester of pregnancy (13–26 weeks)*							
No anemia (≥ 10.5 gm/dL)	61.15	53.88	58.84	55.90	**−8.58**	**−0.333**	0.734
Mild anemia (9.5–10.4 gm/dL)	19.06	24.81	22.58	23.06	**20.98**	**0.333**	0.734
Moderate anemia (7.0–9.4 gm/dL)	17.80	19.11	17.20	19.51	**9.64**	**0.333**	0.734
Severe anemia (< 7.0 gm/dL)	1.94	2.07	1.34	1.46	*−24.87*	*−0.333*	0.734

*Third trimester of pregnancy (27 weeks to delivery)*							
No anemia (≥ 11.0 gm/dL)	42.60	34.13	45.31	43.68	*2.53*	*0.333*	0.734
Mild anemia (10.0–10.9 gm/dL)	22.09	23.76	23.73	23.94	**8.35**	**0.667**	0.308
Moderate anemia (7.0–9.9 gm/dL)	31.93	38.75	29.38	30.76	*−3.67*	*−0.333*	0.734
Severe anemia (< 7.0 gm/dL)	3.31	2.70	1.55	1.61	*−51.34*	*−0.667*	0.308

*Note:* Font style indicates positive change (italic) and negative change (bold) from 1998–1999 to 2019–2021.

## Data Availability

The data that support the findings of this study are available in DHS at https://dhsprogram.com/data/available-datasets.cfm. These data were derived from the following resources available in the public domain: DHS website, https://dhsprogram.com/data/available-datasets.cfm.

## Supporting Information

Supporting Information 1 Supporting File 1 presents distribution of Hb concentration among study population from 1998–1999 to 2019–2021.

Supporting Information 2 Supporting File 2 provides state-wise point estimates of anemia prevalence among pregnant and nonpregnant women with 95% CIs for each survey round.
