# Supplementary material for: Trends in Anemia Prevalence Among Indian Women Using Revised WHO Hemoglobin Cutoffs: Insights From Repeated Cross-Sectional Surveys (1998–2019)
Source: Anemia. 2025 Jul 2;2025:5214630. doi: 10.1155/anem/5214630 (PMC12240656; doi:10.1155/anem/5214630)
Supplement: Supporting Information 1 — Supporting File 1 presents distribution of Hb concentration among study population from 1998–1999 to 2019–2021. [file 5214630.f1.docx]

**Supplementary file 1**: Distribution of Hb concentration amongst study population from 1998-99 to 2019-21

f

e

d

c

b

a
