# Supplementary material for: Trends in Anemia Prevalence Among Indian Women Using Revised WHO Hemoglobin Cutoffs: Insights From Repeated Cross-Sectional Surveys (1998–2019)
Source: Anemia. 2025 Jul 2;2025:5214630. doi: 10.1155/anem/5214630 (PMC12240656; doi:10.1155/anem/5214630)
Supplement: Supporting Information 2 — Supporting File 2 provides state-wise point estimates of anemia prevalence among pregnant and nonpregnant women with 95% CIs for each survey round. [file 5214630.f2.docx]

**Supplementary file 2**: State wise point estimates of anemia prevalence amongst pregnant and non-pregnant women with 95% CIs for each survey round

| State/ UT | Proportion of anemia amongst non-pregnant women (2019-21) | | |  | State/ UT | Proportion of anemia amongst pregnant women (2019-21) | | |
| --- | --- | --- | --- | --- | --- | --- | --- | --- |
|  | Proportion | 95% CI | |  |  | Proportion | 95% CI | |
| Jammu & Kashmir | 0.68 | 0.68 | 0.69 |  | Jammu & Kashmir | 0.39 | 0.34 | 0.44 |
| Himachal Pradesh | 0.54 | 0.53 | 0.55 |  | Himachal Pradesh | 0.34 | 0.25 | 0.44 |
| Punjab | 0.59 | 0.58 | 0.60 |  | Punjab | 0.45 | 0.38 | 0.52 |
| Chandigarh | 0.60 | 0.56 | 0.63 |  | Chandigarh | 0.60 | 0.20 | 0.90 |
| Uttarakhand | 0.40 | 0.39 | 0.40 |  | Uttarakhand | 0.27 | 0.20 | 0.35 |
| Haryana | 0.61 | 0.60 | 0.61 |  | Haryana | 0.46 | 0.40 | 0.52 |
| Delhi | 0.50 | 0.49 | 0.51 |  | Delhi | 0.32 | 0.23 | 0.42 |
| Rajasthan | 0.55 | 0.55 | 0.56 |  | Rajasthan | 0.43 | 0.39 | 0.47 |
| Uttar Pradesh | 0.50 | 0.50 | 0.51 |  | Uttar Pradesh | 0.35 | 0.32 | 0.37 |
| Bihar | 0.65 | 0.64 | 0.65 |  | Bihar | 0.53 | 0.49 | 0.57 |
| Sikkim | 0.41 | 0.39 | 0.42 |  | Sikkim | 0.45 | 0.25 | 0.66 |
| Arunachal Pradesh | 0.39 | 0.39 | 0.40 |  | Arunachal Pradesh | 0.26 | 0.22 | 0.32 |
| Nagaland | 0.30 | 0.29 | 0.31 |  | Nagaland | 0.22 | 0.16 | 0.30 |
| Manipur | 0.29 | 0.28 | 0.30 |  | Manipur | 0.22 | 0.16 | 0.30 |
| Mizoram | 0.37 | 0.36 | 0.38 |  | Mizoram | 0.24 | 0.16 | 0.35 |
| Tripura | 0.67 | 0.66 | 0.68 |  | Tripura | 0.52 | 0.40 | 0.65 |
| Meghalaya | 0.55 | 0.55 | 0.56 |  | Meghalaya | 0.35 | 0.29 | 0.41 |
| Assam | 0.66 | 0.66 | 0.67 |  | Assam | 0.43 | 0.37 | 0.48 |
| West Bengal | 0.72 | 0.72 | 0.73 |  | West Bengal | 0.54 | 0.45 | 0.62 |
| Jharkhand | 0.66 | 0.66 | 0.67 |  | Jharkhand | 0.49 | 0.44 | 0.55 |
| Odisha | 0.64 | 0.64 | 0.65 |  | Odisha | 0.50 | 0.44 | 0.56 |
| Chhattisgarh | 0.64 | 0.63 | 0.64 |  | Chhattisgarh | 0.49 | 0.43 | 0.54 |
| Madhya Pradesh | 0.55 | 0.55 | 0.56 |  | Madhya Pradesh | 0.40 | 0.36 | 0.45 |
| Gujarat | 0.65 | 0.64 | 0.65 |  | Gujarat | 0.55 | 0.48 | 0.61 |
| Dadar and ngar haveli And Daman & Diu | 0.58 | 0.56 | 0.60 |  | Dadar and Nagar haveli And Daman & Diu | 0.33 | 0.18 | 0.54 |
| Maharashtra | 0.55 | 0.55 | 0.56 |  | Maharashtra | 0.36 | 0.31 | 0.42 |
| Andhra Pradesh | 0.59 | 0.58 | 0.60 |  | Andhra Pradesh | 0.41 | 0.29 | 0.54 |
| Karnataka | 0.49 | 0.48 | 0.49 |  | Karnataka | 0.39 | 0.33 | 0.46 |
| Goa | 0.38 | 0.36 | 0.41 |  | Goa | 0.25 | 0.08 | 0.55 |
| Lakshadweep | 0.26 | 0.24 | 0.29 |  | Lakshadweep | 0.08 | 0.01 | 0.39 |
| Kerala | 0.36 | 0.35 | 0.37 |  | Kerala | 0.19 | 0.13 | 0.28 |
| Tamil Nadu | 0.54 | 0.53 | 0.54 |  | Tamil Nadu | 0.39 | 0.32 | 0.46 |
| Puducherry | 0.49 | 0.47 | 0.51 |  | Puducherry | 0.19 | 0.08 | 0.38 |
| Andaman & Nicobar Islands | 0.54 | 0.52 | 0.56 |  | Andaman & Nicobar Islands | 0.67 | 0.38 | 0.87 |
| Telangana | 0.59 | 0.58 | 0.59 |  | Telangana | 0.45 | 0.38 | 0.52 |
| Ladakh | 0.94 | 0.93 | 0.95 |  | Ladakh | 0.88 | 0.75 | 0.94 |

| State/ UT | Proportion of non-pregnant women with anemia (2015-16) | | |  | State/ UT | Proportion of pregnant women with anemia (2015-16) | | |
| --- | --- | --- | --- | --- | --- | --- | --- | --- |
|  | Proportion | 95% CI | |  |  | Proportion | 95% CI | |
| Andaman & Nicobar Islands | 0.63 | 0.61 | 0.65 |  | Andaman & Nicobar Islands | 0.57 | 0.35 | 0.76 |
| Andhra Pradesh | 0.60 | 0.59 | 0.61 |  | Andhra Pradesh | 0.38 | 0.26 | 0.51 |
| Arunachal Pradesh | 0.40 | 0.39 | 0.41 |  | Arunachal Pradesh | 0.24 | 0.18 | 0.30 |
| Assam | 0.46 | 0.45 | 0.47 |  | Assam | 0.29 | 0.24 | 0.34 |
| Bihar | 0.61 | 0.60 | 0.61 |  | Bihar | 0.48 | 0.44 | 0.51 |
| Chandigarh | 0.77 | 0.73 | 0.80 |  | Chandigarh | 0.67 | 0.10 | 0.97 |
| Chhattisgarh | 0.49 | 0.49 | 0.50 |  | Chhattisgarh | 0.36 | 0.31 | 0.41 |
| Dadra And Nagar Haveli | 0.80 | 0.77 | 0.83 |  | Dadra And Nagar Haveli | 0.43 | 0.20 | 0.69 |
| Daman And Diu | 0.57 | 0.55 | 0.60 |  | Daman And Diu | 0.17 | 0.02 | 0.68 |
| Goa | 0.32 | 0.29 | 0.34 |  | Goa | 0.31 | 0.12 | 0.60 |
| Gujarat | 0.57 | 0.56 | 0.57 |  | Gujarat | 0.43 | 0.37 | 0.49 |
| Haryana | 0.63 | 0.62 | 0.63 |  | Haryana | 0.50 | 0.46 | 0.55 |
| Himachal Pradesh | 0.56 | 0.55 | 0.57 |  | Himachal Pradesh | 0.30 | 0.22 | 0.41 |
| Jammu And Kashmir | 0.52 | 0.52 | 0.53 |  | Jammu And Kashmir | 0.26 | 0.21 | 0.32 |
| Jharkhand | 0.66 | 0.65 | 0.66 |  | Jharkhand | 0.51 | 0.46 | 0.57 |
| Karnataka | 0.46 | 0.45 | 0.46 |  | Karnataka | 0.37 | 0.30 | 0.43 |
| Kerala | 0.34 | 0.33 | 0.35 |  | Kerala | 0.19 | 0.14 | 0.27 |
| Lakshadweep | 0.45 | 0.42 | 0.48 |  | Lakshadweep | 0.25 | 0.08 | 0.57 |
| Madhya Pradesh | 0.54 | 0.53 | 0.54 |  | Madhya Pradesh | 0.44 | 0.41 | 0.47 |
| Maharashtra | 0.47 | 0.46 | 0.47 |  | Maharashtra | 0.40 | 0.34 | 0.46 |
| Manipur | 0.26 | 0.25 | 0.27 |  | Manipur | 0.18 | 0.14 | 0.24 |
| Meghalaya | 0.55 | 0.54 | 0.56 |  | Meghalaya | 0.36 | 0.29 | 0.45 |
| Mizoram | 0.28 | 0.27 | 0.29 |  | Mizoram | 0.20 | 0.14 | 0.28 |
| Nagaland | 0.27 | 0.26 | 0.28 |  | Nagaland | 0.21 | 0.15 | 0.29 |
| Delhi | 0.52 | 0.50 | 0.53 |  | Delhi | 0.39 | 0.27 | 0.53 |
| Odisha | 0.54 | 0.54 | 0.55 |  | Odisha | 0.41 | 0.36 | 0.46 |
| Puducherry | 0.48 | 0.46 | 0.49 |  | Puducherry | 0.24 | 0.12 | 0.41 |
| Punjab | 0.53 | 0.52 | 0.54 |  | Punjab | 0.33 | 0.27 | 0.39 |
| Rajasthan | 0.48 | 0.48 | 0.49 |  | Rajasthan | 0.36 | 0.32 | 0.40 |
| Sikkim | 0.37 | 0.35 | 0.38 |  | Sikkim | 0.18 | 0.09 | 0.31 |
| Tamil Nadu | 0.55 | 0.55 | 0.56 |  | Tamil Nadu | 0.34 | 0.28 | 0.41 |
| Tripura | 0.55 | 0.53 | 0.56 |  | Tripura | 0.34 | 0.20 | 0.51 |
| Uttar Pradesh | 0.53 | 0.52 | 0.53 |  | Uttar Pradesh | 0.37 | 0.35 | 0.40 |
| Uttarakhand | 0.44 | 0.43 | 0.45 |  | Uttarakhand | 0.32 | 0.26 | 0.38 |
| West Bengal | 0.63 | 0.63 | 0.64 |  | West Bengal | 0.50 | 0.42 | 0.58 |
| Telangana | 0.58 | 0.56 | 0.59 |  | Telangana | 0.52 | 0.41 | 0.63 |

| State/ UT | Proportion of anemia amongst non-pregnant women (2005-06) | | |  | State/ UT | Proportion of anemia amongst pregnant women (2005-06) | | |
| --- | --- | --- | --- | --- | --- | --- | --- | --- |
|  | Proportion | 95% CI | |  |  | Proportion | 95% CI | |
| Jammu & Kashmir | 0.52 | 0.50 | 0.54 |  | Jammu & Kashmir | 0.43 | 0.27 | 0.61 |
| Himachal Pradesh | 0.42 | 0.40 | 0.44 |  | Himachal Pradesh | 0.18 | 0.08 | 0.36 |
| Punjab | 0.38 | 0.36 | 0.40 |  | Punjab | 0.26 | 0.15 | 0.41 |
| Uttaranchal | 0.55 | 0.53 | 0.57 |  | Uttaranchal | 0.50 | 0.33 | 0.67 |
| Haryana | 0.55 | 0.53 | 0.57 |  | Haryana | 0.64 | 0.47 | 0.78 |
| Delhi | 0.46 | 0.44 | 0.48 |  | Delhi | 0.21 | 0.10 | 0.37 |
| Rajasthan | 0.52 | 0.51 | 0.54 |  | Rajasthan | 0.53 | 0.39 | 0.66 |
| Uttar Pradesh | 0.48 | 0.47 | 0.49 |  | Uttar Pradesh | 0.36 | 0.30 | 0.43 |
| Bihar | 0.68 | 0.66 | 0.69 |  | Bihar | 0.46 | 0.36 | 0.56 |
| Sikkim | 0.58 | 0.56 | 0.60 |  | Sikkim | 0.38 | 0.20 | 0.60 |
| Arunachal Pradesh | 0.51 | 0.48 | 0.53 |  | Arunachal Pradesh | 0.33 | 0.13 | 0.62 |
| Manipur | 0.36 | 0.35 | 0.38 |  | Manipur | 0.22 | 0.13 | 0.34 |
| Mizoram | 0.38 | 0.36 | 0.40 |  | Mizoram | 0.48 | 0.29 | 0.68 |
| Tripura | 0.66 | 0.64 | 0.68 |  | Tripura | 0.53 | 0.29 | 0.76 |
| Meghalaya | 0.44 | 0.42 | 0.47 |  | Meghalaya | 0.52 | 0.34 | 0.69 |
| Assam | 0.69 | 0.67 | 0.70 |  | Assam | 0.57 | 0.43 | 0.71 |
| West Bengal | 0.60 | 0.59 | 0.62 |  | West Bengal | 0.50 | 0.37 | 0.63 |
| Jharkhand | 0.68 | 0.66 | 0.69 |  | Jharkhand | 0.57 | 0.42 | 0.70 |
| Orissa | 0.60 | 0.59 | 0.62 |  | Orissa | 0.49 | 0.35 | 0.63 |
| Chhattisgarh | 0.56 | 0.54 | 0.58 |  | Chhattisgarh | 0.49 | 0.34 | 0.63 |
| Madhya Pradesh | 0.51 | 0.50 | 0.52 |  | Madhya Pradesh | 0.46 | 0.35 | 0.57 |
| Gujarat | 0.55 | 0.54 | 0.57 |  | Gujarat | 0.52 | 0.39 | 0.64 |
| Maharashtra | 0.49 | 0.47 | 0.50 |  | Maharashtra | 0.42 | 0.31 | 0.53 |
| Andhra Pradesh | 0.59 | 0.57 | 0.60 |  | Andhra Pradesh | 0.40 | 0.28 | 0.53 |
| Karnataka | 0.51 | 0.50 | 0.52 |  | Karnataka | 0.58 | 0.44 | 0.71 |
| Goa | 0.38 | 0.36 | 0.40 |  | Goa | 0.26 | 0.11 | 0.50 |
| Kerala | 0.33 | 0.31 | 0.34 |  | Kerala | 0.23 | 0.12 | 0.39 |
| Tamil Nadu | 0.53 | 0.51 | 0.54 |  | Tamil Nadu | 0.39 | 0.26 | 0.54 |

| State/ UT | Proportion of anemia amongst non-pregnant women (1998-99) | | |  | State/ UT | Proportion of anemia amongst pregnant women (1998-99) | | |
| --- | --- | --- | --- | --- | --- | --- | --- | --- |
|  | Proportion | 95% CI | |  |  | Proportion | 95% CI | |
| Andhra Pradesh | 0.50 | 0.48 | 0.52 |  | Andhra Pradesh | 0.26 | 0.15 | 0.42 |
| Assam | 0.69 | 0.67 | 0.71 |  | Assam | 0.49 | 0.38 | 0.61 |
| Bihar | 0.65 | 0.63 | 0.66 |  | Bihar | 0.35 | 0.28 | 0.43 |
| Goa | 0.37 | 0.34 | 0.39 |  | Goa | 0.29 | 0.13 | 0.51 |
| Gujarat | 0.46 | 0.44 | 0.48 |  | Gujarat | 0.28 | 0.17 | 0.41 |
| Haryana | 0.46 | 0.45 | 0.48 |  | Haryana | 0.44 | 0.33 | 0.56 |
| Himachal Pradesh | 0.33 | 0.31 | 0.35 |  | Himachal Pradesh | 0.21 | 0.12 | 0.35 |
| Jammu & Kashmir | 0.48 | 0.46 | 0.50 |  | Jammu & Kashmir | 0.35 | 0.25 | 0.48 |
| Karnataka | 0.42 | 0.41 | 0.44 |  | Karnataka | 0.31 | 0.20 | 0.43 |
| Kerala | 0.23 | 0.21 | 0.24 |  | Kerala | 0.07 | 0.02 | 0.25 |
| Madhya Pradesh | 0.52 | 0.51 | 0.53 |  | Madhya Pradesh | 0.42 | 0.35 | 0.50 |
| Maharashtra | 0.46 | 0.45 | 0.47 |  | Maharashtra | 0.42 | 0.32 | 0.53 |
| Manipur | 0.26 | 0.24 | 0.29 |  | Manipur | 0.20 | 0.09 | 0.38 |
| Meghalaya | 0.61 | 0.57 | 0.65 |  | Meghalaya | 0.43 | 0.26 | 0.61 |
| Mizoram | 0.45 | 0.42 | 0.48 |  | Mizoram | 0.35 | 0.19 | 0.54 |
| Nagaland | 0.32 | 0.28 | 0.35 |  | Nagaland | 0.14 | 0.05 | 0.36 |
| Orissa | 0.62 | 0.61 | 0.64 |  | Orissa | 0.42 | 0.31 | 0.54 |
| Punjab | 0.42 | 0.40 | 0.44 |  | Punjab | 0.32 | 0.22 | 0.44 |
| Rajasthan | 0.48 | 0.47 | 0.50 |  | Rajasthan | 0.43 | 0.37 | 0.50 |
| Sikkim | 0.48 | 0.45 | 0.52 |  | Sikkim | 0.11 | 0.02 | 0.50 |
| Tamil Nadu | 0.55 | 0.54 | 0.57 |  | Tamil Nadu | 0.31 | 0.20 | 0.44 |
| West Bengal | 0.62 | 0.61 | 0.64 |  | West Bengal | 0.57 | 0.43 | 0.70 |
| Uttar Pradesh | 0.48 | 0.47 | 0.50 |  | Uttar Pradesh | 0.34 | 0.26 | 0.42 |
| New Delhi | 0.41 | 0.39 | 0.43 |  | New Delhi | 0.27 | 0.16 | 0.42 |
| Arunachal Pradesh | 0.57 | 0.54 | 0.60 |  | Arunachal Pradesh | 0.40 | 0.27 | 0.55 |
| Tripura | 0.59 | 0.55 | 0.62 |  | Tripura | 0.38 | 0.18 | 0.62 |
